# Multiplex PCR Screening of Y-chromosome microdeletions in azoospermic ICSI candidate men

**Published:** 2013-04

**Authors:** Mohammad Hasan Sheikhha, Mohammad Ali Zaimy, Saeede Soleimanian, Seyed Mehdi Kalantar, Azam Rasti, Maryam Golzade, Hamid Hoseini Fahraji

**Affiliations:** 1 *Research and Clinical Center for Infertility, Shahid Sadoughi University of Medical Sciences, Yazd, Iran.*; 2 *Department of Animal Sciences, Agriculture Faculty, Urumia University, Urumia, Iran. *

**Keywords:** *Male infertility*, *Multiplex PCR*, *Y chromosome microdeletion*

## Abstract

**Background: **It has been hypothesized that Y-q microdeletion can account for significant proportion of infertility in men. There are three nonoverlapping regions referred to as the "azoozpermia factors" AZFa, AZFb, and AZFc from proximal to distal part of Y-q. These have been defined as spermatogenesis loci, this region deletions have been shown to be involved in male azoospermic or severe oligoozospermic infertility.

**Objective:** Evaluation the rate of Y-chromosome microdeletions in infertile men.

**Materials and Methods: **In this case-control study, 25 azoospermic infertile men candidate for intracytoplasmic sperm injection (ICSI) were selected as case group. For control group, 25 normoozoospemric men were selected. All cases and controls had normal 46XY karyotype. DNA extraction and molecular analysis were done on blood samples. Multiplex-PCR method was done to identify the presence of microdeletion in AZFa, AZFb or AZFc loci. Eight STS primers that include two controls were selected to determine Y-chromosome microdeletions.

**Results:** 20% (5/25) of all patients have at least one microdeletion in more than one region of AZF loci. Totally 17 microdeletions was observed, one case had deletions in three AZF regions, and 4 cases had deletions in two AZF regions. The rate of deletions was 42% (7/17) for AZFc, 35% (6/17) for AZFa and 23% (4/17) for AZFb.

**Conclusion:** The molecular DNA analysis could help us to know the real cause of infertility and can give good information for good decision for example in men whit microdeletions who want to undertake ICSI procedure the deletions will be passed to their son.

## Introduction

It is estimated that around 15% of the couple at reproductive age are infertile, and approximately half of the infertilities are caused by male factor (1). Defective spermatogenesis is the result of several different disorders, such as endocrinological disorders, malnutrition, genetic defects and maybe environmental condition (2). Around 10% of males with azoospermia and oligozoospermia, have interstitial microdeletion on the Y-chromosome (3-5). 

Male-related genes including sex-determining region of Y-chromosome (SRY) and several spermatogenesis-related genes are accumulated in Y chromosome (6). The AZFa, AZFb and AZFc are three major candidate regions of azoospermic factor on long arm of Y-chromosome. These regions have several candidate for the factor: AZFa; USP9Y and DBY that encode an ubiquitin specific protease and a RNA-helicase respectively; AZFb contains several candidate genes such as RBMY, encode an RAN-binding protein; and AZFc contains DAZ and CDY family (5, 7-11). 

Current analysis of human genome has showed that the Y-chromosome long arm has many palindromes or inverted repeats. This typical structure is presumed to cause the microdeletion in AZF regions found in some of infertile men (11-13). There are 300 sequence tagged sites (STS) in the Y-chromosome that mapped for the above three AZF regions (14). This study aim was to calculate the frequency of AZF microdeletions among azoospermic intracytoplasmic sperm injection (ICSI) candidate men that attended to Yazd Research and Clinical Center for Infertility.

## Materials and methods

In this case-control study, 25 infertile men attending to Yazd Research and Clinical Center for Infertility who were candidate for intracytoplasmic sperm injection (ICSI) were selected randomly in 2011. All of the selected patients were azoospermic with normal 46XY karyotype and had a positive history of male factor infertility. The control group consists of 25 normozoospermic men with normal karyotype. The Ethical Committee of Yazd Research and Clinical Center for Infertility approved the study and all of the participants consented to enter this study verbally. Inclusion criteria were 25-40 year-old men with azoospermia and exclusion criteria was azoospermic men with obstructive tract or any chromosomal abnormality. DNA was extracted from leukocytes of peripheral blood samples by a salting out method (15). 

A series of 6 STS markers on the long arm of Y-chromosome were used for detection of interstitial microdeletions according to the European Academy of Andrology (EAA), the European Molecular Genetic Quality network (EMQN) and other protocols. The markers consisted of sY84 and Sy86 for AZFa, sY127 and sY134 for AZFb, sY254 and sY255 for AZFc regions. The sequence and size of all of primers are shown in table I.

**Multiplex-PCR**

DNA amplified by multiplex PCR method. Two sets of amplification reactions were used. In each PCR 3 STS primers including two internal controls were used. Primers of each reactions had similar melting temperature (Tm) (Table II). PCR amplification condition had a thermocycling procedure consisted of 4min in 94^o^C for initial denaturation. The procedure followed by 32 cycles of 30s at 94^o^C, 30s at 59^o^C and 4min in 65^o^C with a final extension at 65^o^C for 5min.

**Gel electrophoresis**

The products of PCR were run by electrophoresis on a 2% agarose gel.

**Statistical analysis**

X^2^ test was carried out to compare difference between the cases and controls. The statistical analyses were performed with SPSS 16 statistical software when p-value was under 0.05 the difference was considered significant.

## Results

A total of 25 infertile men who were candidate for ICSI wereselected. The average age in case group was 30.5 years (26-36.5 years) and in control group was 28 years (25-34 years). Of these patients, 5 cases have deletions in more than one region of AZF loci on Y-chromosome but no microdeletion was detected among control group. Totally 17 microdeletions was observed. Among the regions, AZFc had the most microdeletions 42% (7/17) followed by AZFa 35% (6/17) and the AZFb microdeletions have the less frequency 23% (4/17). In this study, among patients with microdeletion one case had deletions in all three AZF regions. Four cases had deletions in two regions and two cases had deletion just in one region. In total, 16% of all cases have deletions in AZFa, 16% in AZFc and 12% in AZFb regions.

**Table I:** Primers sequence and size

	**STS**	**Base pairs**	**primers**
	ZFY	495	5´-ACC RCT GTA CTG ACT GTG ATT ACA C-3´
			5´-GCA CYT CTT TGG TAT CYG AGA AAG T-3´
	SRY	472	5´-GAA TAT TCC CGC TCT CCG GA-3´
			5´-GCT GGT GCT CCA TTC TTG AG-3´
AZFa	sY84	326	5´-AGA AGG GTC TGA AAG CAG GT-3´
			5´-GCC TAC CTG GAG GAG GCT TC-3´
AZFa	sY86	320	5´-GTG ACA CAC AGA CTA TGC TTC-3´
			5´-ACA CAC AGA GGG ACA ACC CT-3´
AZFb	sY127	274	5´-GGC TCA CAA ACG AAA AGA AA-3´
			5´-CTG CAG GCA GTA ATA AGG GA-3´
AZFb	Sy134	301	5´-GTC TGC CTC ACC ATA AAA CG-3´
			5´-CCG TGT GCT GGA GAC TAA TC-3´
AZFc	sY254	380	5´-GGG TGT TAC CAG AAG GCA AA-3´
			5´-GAC CGT ATC TAC CAA AGC TGC-3´
AZFc	sY255	126	5´-GTT ACA GGA TTC GGC GTG AT-3´
			5´-CTC GTC ATG TGC AGC CAC-3´

STS: sequence tagged sites

**Table II:** Primer mix of each Multiplex-PCR reaction

**Reaction A**		**Reaction B**	
ZFY	495bp	ZFY	495bp
SRY	472bp	SRY	472bp
sY86	326bp	sY84	320bp
sY134	301bp	sY127	274bp
sY255	126bp	sY254	400bp

**Table III:** Results of different studies

	**n**	**AZFc (%)**	**AZFb (%)**	**AZFa (%)**
AM Malek Asgar (2008)	50	44	4	12
Junjicmy Fe et al (2002)	73	0	1.36	12.3
Roy A. Brandell et al (1998)	80	1.25	8.75	7.5
SJ Silber et al (1998)	51	0	0	19.6
Jon C. Rroyor et al (1997)	20	10	10	10
JYM TSE et al (2000)	35	0	0	8.6
Sarah K. Girandi et al (1997)	108	0	4.6	3.7
Martinez et al (2000)	57	0	8.8	12.3
Kleamn et al (1999)	105	0.95	1.9	5.7
Fujisama et al (2001)	54	3.7	16.7	18.5
Present study	25	16	12	16

## Discussion

Deletions of AZF regions are deletions of the euchromatine part of the Y chromosome long arm. It is assumed that deletions of this part of Y chromosome can damage genes in this region that is responsible for the proper course of spermatogenesis. Many factors including somatic and sex chromosome genes interaction candidate to the normal spermatogenesis and the AZF deletions are the most frequent cause of spermatogenetic failure (6). 

After Klinefelter’s syndrome, Y-chromosomal deletions are the second most frequent spermatogenesis disorder in infertile men. In the last few years some of research and clinical institutions have described screening of Y chromosome microdeletions in infertile men and molecular diagnostics of this type of Y-chromosomal disorders has become an important diagnostic test within laboratories worldwide dealing with these disorders (16-18). 

Y chromosome AZF regions microdeletions are frequently found in azoospermic patients. Deletions incidence has been found from 3-55% (19, 20). In table III we compared our results whit other similar studies. EAA and EMQN published the guidelines for molecular screening of Y-chromosome microdeletions (21). In this study we used six sequences according to European guidelines in which all three sub-regions are represented by Y sequences: sY84, sY86, sY127, sY134, sY254 and sY255. Whit use of these STSs we reported 16% of microdeletions in AZFa, 12% in AZFb and 16% in AZFc regions. 

The deletions in an infertile man could provide a proper understanding of the disease allows the medical stuff to avoid unnecessary expensive treatment to fertility improvement. Microdeletions of azoospermia factors are characteristic for spermatogenic failures and lead to oligozoospermia or azoospermia. PCR analysis of these deletions helps to determine site and the frequency of gene deletion and presents a defined prognosis and valuable counseling for couple whit fertility disorders. In the patients' with microdeletions that want to undertake ICSI procedure the deletions may be passed to their son. Because of this probability ethical consequences should be important dimensions of this technique. 
